# Molecular Understanding of the Role of Catalyst Particle Arrangement in Local Mass Transport Resistance for Fuel Cells

**DOI:** 10.1002/advs.202409755

**Published:** 2024-12-15

**Authors:** Aoxin Ran, Linhao Fan, Chasen Tongsh, Jiaqi Wang, Zhengguo Qin, Qing Du, Meng Ni, Kui Jiao

**Affiliations:** ^1^ State Key Laboratory of Engines Tianjin University Tianjin 300200 China; ^2^ Department of Building and Real Estate Research Institute for Sustainable Urban Development (RISUD) & Research Institute for Smart Energy (RISE) Hong Kong Polytechnic University Hong Kong 100872 China; ^3^ National Industry‐Education Platform for Energy Storage Tianjin University Tianjin 300200 China

**Keywords:** dispersity, fuel cells, interparticle distance, oxygen transport, uniformity

## Abstract

Platinum (Pt) catalyst performance loss caused by a high local oxygen transport resistance is an urgent problem to be solved for proton exchange membrane fuel cells (PEMFCs). Rationally arranging Pt particles on carbon support is the primary approach for reducing mass transport resistance. Herein, using a unique method coupling Hybrid Reverse Monte Carlo, molecular dynamics simulations, and experimental measurements, a Pt particle arrangement strategy is proposed to reduce local oxygen transport resistance, based on a molecular‐level understanding of its impact. The densely arranged Pt particles with a small interparticle distance lead to the denser ionomer layer due to the co‐attraction effect, leading to a high local oxygen transport resistance. The nonuniformly arranged Pt particles with various interparticle distances cause the heterogeneous ionomer density, inducing the heterogeneous oxygen transport. Increasing the Pt‐Pt interparticle distance from 2 to 5 nm substantially reduces the local oxygen transport resistance by over 50%. The uniform arrangement of Pt particles makes the ionomer layer density more homogeneous, resulting in more uniform oxygen transport. Therefore, uniformly arranging Pt particles with an interparticle distance of >5 nm on carbon support is preferred for reducing local oxygen transport resistance and improving the homogeneity of oxygen transport.

## Graphical abstract

This paper elucidates the influence of the Pt particle arrangement on carbon support on oxygen transport, identifying that a close arrangement of Pt particles results in a co‐attraction effect that hinders oxygen transport. It is therefore recommended that uniformly arranging Pt particles on carbon support with a >5 nm interparticle spacing can avoid the co‐attraction effect, thus improving oxygen transport.

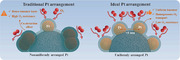

## 1 Introduction

Hydrogen energy is promising to achieve net zero carbon emissions.^[^ ^1^, ^2^ ^]^ Proton exchange membrane fuel cell (PEMFC) is an effective hydrogen utilization technology, which can directly convert the chemical energy in hydrogen and oxygen to electrical energy. Due to its advantages of clean production, high efficiency, and rapid response, PEMFC has been widely used in many fields, such as transportation, power plants, and aerospace.^[^ ^3^, ^4^, ^5^ ^]^ However, the utilization of platinum (Pt) catalysts mainly leads to the high cost of PEMFCs, ≈60 $ kW^−1^ of PEMFC system in 2022 for light‐duty vehicles according to the U.S. Department of Energy.^[^ ^6^, ^7^, ^8^, ^9^ ^]^ The cost of Pt catalysts accounts for 40% of PEMFC stack cost at a production volume of 500 000 per year.^[^ ^6^, ^7^ ^]^ To reduce the PEMFC cost for large‐scale commercialization, the Pt loading in a PEMFC stack is expected to be reduced to <0.1g_pt_ kW^−1^, that is <10 g_pt_ for a 100 kW stack.^[^ ^7^, ^10^ ^]^

To achieve high performance at a low Pt loading, improving the intrinsic catalytic activity is the promising approach. Therefore, many novel catalysts, including nanowires,^[^ ^11^, ^12^ ^]^ nanoframe,^[^ ^13^ ^]^ nanocage,^[^ ^14^ ^]^ core‐shell,^[^ ^15^, ^16^ ^]^ etc., have been developed to even achieve a high mass activity via the rotating disk electrode measurement. For example, the nanowire catalyst developed by Li et al.^[^ ^11^ ^]^ achieved an extremely high mass activity of 13.6 A mg_Pt_^−1^, while the core‐shell catalyst developed by Chong et al.^[^ ^15^ ^]^ achieved a mass activity of 12.36 A mg_Pt_^−1^. However, the mass activities of the nanowire and core‐shell catalysts decreased to 1.06 and 1.77 A mg_Pt_^−1^, respectively, measured in the actual membrane electrode assembly (MEA).^[^ ^12^, ^15^ ^]^ The unexpected activity loss of catalysts is mainly induced by the local oxygen transport resistance in the MEA.^[^ ^17^, ^18^, ^19^, ^20^, ^21^ ^]^ Therefore, both the intrinsic catalytic activity and local oxygen transport resistance highly affect the performance of catalysts in the actual PEMFC application.

In the cathode catalyst layer (CL) of PEMFCs, oxygen molecules permeate through ionomer films to Pt particles supported by the carbon particle for reaction.^[^ ^22^, ^23^, ^24^, ^25^, ^26^ ^]^ Therefore, the arrangement of Pt particles on the carbon support highly affects the ionomer film structures and thus the local oxygen transport resistance. Oxygen transport resistance decreased when increasing the Pt dispersity (i.e., increasing the Pt‐Pt interparticle distance) on the carbon support based on the results from Lee et al.^[^ ^27^ ^]^ and Owejan et al.^[^ ^28^ ^]^ Recently, Islam et al.^[^ ^10^ ^]^ successfully prepared nitrogen‐doping carbon support that achieved the uniform distribution of Pt particles with a large Pt‐Pt interparticle distance. The novel design achieved an unprecedentedly low local oxygen transport resistance for ultra‐low Pt loading (0.034 g_Pt_ cm^−2^) catalyst layer, which is approximately half of the conventional one. Although they deduced from the experimental results that increasing the Pt‐Pt distance mitigated the so‐called territory effect and thus reduced the local oxygen transport resistance, the underlying mechanisms related to the structural properties of ionomer films on carbon‐supported Pt particles and the transport properties of oxygen molecules are still unclear.

The nanoscale oxygen transport near catalysts is difficult or impossible to be experimentally observed. To understand underlying oxygen transport mechanisms, molecular dynamics (MD) simulation has been widely used due to its strong capability in exploring nanoscale transport. Kurihara et al.^[^ ^29^ ^]^ found that the oxygen transport resistance through the ionomer/Pt interface was higher than that the ones through other regions in ionomer films, meaning that the ionomer/Pt interface played a dominant role in the oxygen transport resistance. Fan et al.^[^ ^30^, ^31^ ^]^ found that the ultrathin dense layer close to Pt particles highly blocked the oxygen transport and the region near the edges and corners of Pt particles showed a lower ionomer density to form a low oxygen transport resistance region. Therefore, the major source of oxygen transport resistance was the dense layer in the ionomer/Pt interface based on MD simulations. Suppressing the dense layer is crucial to reduce the oxygen transport resistance.^[^ ^32^, ^33^, ^34^ ^]^ However, the previous MD simulations are certainly unable to explore the oxygen transport near multiple Pt particles, due to considering a Pt surface or single Pt particle in simulations. Therefore, further MD simulations are needed to guide the rational arrangement of multiple Pt particles on the carbon particle in terms of Pt‐Pt distance and Pt uniformity for achieving a low oxygen transport resistance.

**Figure 1:**
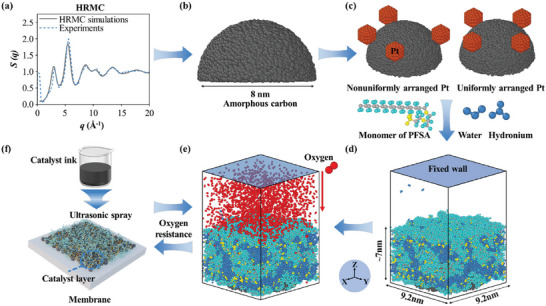
The framework of this study coupling Hybrid Reverse Monte Carlo (HRMC), molecular dynamics (MD) simulations, and experiments. a) Structural factor *S* (*q*) obtained by HRMC simulations and experiments;^[^ ^35^ ^]^ b) amorphous carbon; c) Pt particles uniformly and nonuniformly arranged on carbon support; d) equilibrium ionomer film on carbon‐supported Pt particles; e) oxygen transport through ionomer film to Pt particles; f) preparation of catalyst layer by ultrasonic spraying. The gray, cyan, yellow, blue, red, orange, and black beads represent the carbon atoms of PFSA, fluorine atoms of PFSA, sulfur/oxygen atoms of PFSA, water molecules/hydroniums, oxygen molecules, Pt atoms, and carbon atoms of amorphous carbon, respectively.

Herein, we perform the well‐designed MD simulations to investigate the role of Pt catalyst particle arrangement in local oxygen transport resistance through ionomer films on carbon‐supported Pt particles, considering the real amorphous carbon obtained by Hybrid Reverse Monte Carlo (HRMC) method, multiple Pt particles, and perfluorosulfonic acid (PFSA) ionomer films as shown in **Figure** **1**. Experimental measurements of oxygen transport resistances are carried out to verify MD simulations. The structural properties of ionomer film and oxygen transport properties near carbon‐supported Pt particles are investigated. The effects of Pt particle dispersity and uniformity on the oxygen transport resistance through ionomer films are revealed. Subsequently, we propose a superior arrangement strategy of Pt particles on carbon support to reduce local oxygen transport resistance.

## 2 Results and Discussion

### 2.1 Ionomer Film Structures

**Figure 2:**
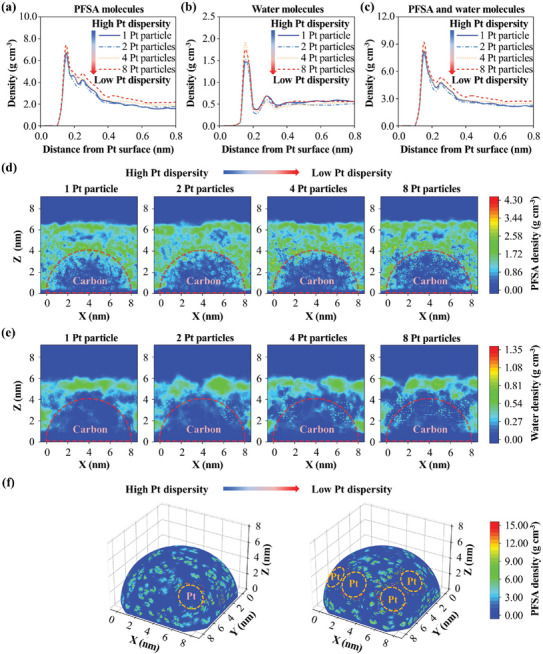
1D density distributions of a) PFSA molecules, b) water molecules, and c) all molecules around nonuniformly arranged Pt particles; 2D density distributions of d) PFSA molecules and e) water molecules on the X‐Z plane; and f) 3D density of PFSA molecules for nonuniformly arranged Pt particles.

The ionomer film structures on carbon‐supported Pt particles are first investigated in this subsection. **Figure** **2** shows the 1D density distributions of PFSA and water molecules at different distances from the Pt surface, while Figure S1 (Supporting Information) shows the 1D density distributions at different distances from the carbon center. It is noteworthy that the fewer Pt particles on the carbon support mean the higher Pt particle dispersity. As shown in Figure 2a,b, the density of PFSA and water molecules in ionomer films increases with the decrease of Pt particle dispersity on the carbon support, thereby increasing the total density of ionomer films as shown in Figure 2c, due to the stronger attraction of Pt particles with a small interparticle distance to PFSA and water molecules. Moreover, as shown in Figure S1 (Supporting Information), the water density on the carbon support increases as the Pt particle dispersity decreases, which means that the carbon‐supported Pt particle surface becomes more hydrophilic at a low Pt dispersity, agreeing with previous experimental works.^[^ ^36^, ^37^, ^38^ ^]^ The density distributions for uniformly arranged Pt particles on the carbon support in Figure S2 (Supporting Information) show similar results.

Moreover, the 2D density distributions of PFSA and water molecules are also obtained and shown in Figure 2d,e. The red lines in those figures represent the location of carbon particles. The locations of Pt particles and carbon support on the X‐Z plane are shown in Figure S3 (Supporting Information). Obviously, the carbon surface with a lower Pt particle dispersity has a lower PFSA density and higher water density. To observe the PFSA ionomer distribution near Pt particles, the PFSA density distributions at 5.5 nm from the center of carbon particles are obtained and shown in Figure 2f. The PFSA density near the densely arranged Pt particles with a lower dispersity is higher due to the co‐attraction of Pt particles to the long PFSA chains, which is also observed in the snapshots of Figure S4 (Supporting Information). Due to the strong attraction of Pt particles, the PFSA density close to the carbon particle decreases when the Pt particle dispersity decreases, while the water density increases. The density distributions for uniformly arranged Pt particles on the carbon support in Figure S5 (Supporting Information) show similar results. Therefore, the small Pt‐Pt interparticle distance under a low dispersity causes a tight arrangement of PFSA chains on Pt particles, which will highly block the oxygen transport to Pt particles.

### 2.2 Oxygen Transport

**Figure 3:**
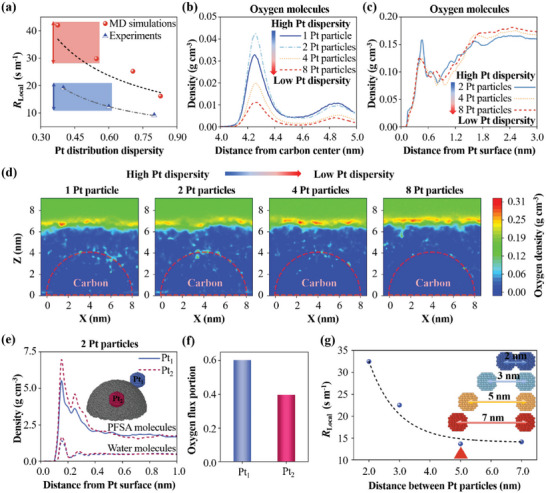
a) Oxygen transport resistances (*R*_Local_) obtained by MD simulations and experiments, 1D density distributions of oxygen molecules at different distances from the b) carbon center and c) Pt surface; d) 2D density distributions of oxygen molecules on the X‐Z plane; e) 1D density distributions of PFSA molecules and water molecules at different distances from each Pt particle surface and f) oxygen transport flux portions to each Pt particle (Pt_1_ and Pt_2_); and g) oxygen transport resistances (*R*_Local_) to the 2 nm Pt particles with different interparticle distances.

During MD simulations, the number of oxygen molecules that reach Pt particles is recorded and plotted in Figure S6 (Supporting Information), which shows that the oxygen number increases almost linearly with increasing simulation time, meaning that the oxygen transport process reaches a steady state. Then the oxygen transport resistances through ionomer films for different Pt dispersity are calculated based on Equations (6) and (7). Moreover, the oxygen transport resistances are also measured using the limiting current method based on the prepared CLs. More detailed test data are shown in Figure S7 (Supporting Information). As shown in **Figure** **3**a, the oxygen transport resistance at a lower Pt particle dispersity is higher despite the thinner CL, which demonstrates that the oxygen transport resistance through the ionomer film increases with the decrease of Pt particle dispersity. The MD simulation results show a similar trend, which also agrees with the results by Lee et al.^[^ ^27^ ^]^ and Owejan et al.^[^ ^28^ ^]^

To understand underlying oxygen transport mechanisms, the oxygen density distributions in ionomer films are analyzed. First, the 1D density distributions of oxygen molecules at different distances from the carbon center and Pt surface are shown in Figure 3b,c, respectively. A higher oxygen density peak is observed near the Pt surface and carbon surface. Moreover, the density peak is higher when the Pt particle dispersity is higher. Furthermore, the 2D density distributions of oxygen molecules on the X‐Z plane are shown in Figure 3d. Obviously, the density of oxygen molecules on carbon‐supported Pt particles with a higher dispersity is higher, thereby leading to a lower oxygen transport resistance shown in Figure 3a. The tight arrangement of PFSA chains on carbon‐supported Pt particles with a low dispersity shown in Figure 2f limits the migration of oxygen molecules, leading to a lower oxygen density and higher oxygen transport resistance. The oxygen density distributions for uniformly arranged Pt particles on the carbon support in Figure S8 (Supporting Information) show similar results. Furthermore, the ionomer densities and oxygen transport fluxes for each Pt particle for the system with two Pt particles are evaluated. The 1D density distributions of PFSA and water molecules at different distances from each Pt particle (Pt_1_ and Pt_2_) surface are shown in Figure 3e. The PFSA and water density are both higher near the Pt_1_ particle than the Pt_2_ particle. In consequence, the oxygen transport flux to the Pt_1_ particle is lower than that to the Pt_2_ particle as shown in Figure 3f, which again demonstrates that the dense ionomer causes the high oxygen transport resistance. Moreover, the oxygen transport resistances to each Pt particle on the carbon support are different due to the heterogeneous ionomer density around them. In addition, the color‐filled contours in Figure S9 (Supporting Information) represent the density distributions of PFSA and water molecules, while the black and red lines are the oxygen density contours at 0.04 and 0.1 g cm^−3^, respectively. As shown in Figure S9 (Supporting Information), the PFSA aggregates and water aggregates are both present in the ionomer film. The area with a high oxygen density tends to show the low densities of PFSA and water. Therefore, it can be inferred that the oxygen molecules prefer to migrate via the interface between PFSA aggregates and water aggregates.

As analyzed above, the small Pt‐Pt interparticle distance under a lower dispersity leads to the denser ionomer induced by the co‐attraction of Pt particles, thereby causing a higher oxygen transport resistance. However, too few Pt particles with a larger interparticle distance on an identical carbon particle will thicken the CL with the same Pt loading, which is detrimental to the oxygen transport through the CL. Therefore, it is essential to search for an appropriate Pt‐Pt interparticle distance to balance the oxygen transport resistance and the Pt loading per carbon particle. Inaba et al.^[^ ^39^ ^]^ carried out the experimental studies, which controlled the interparticle distances of ≈1–15 nm. Therefore, we also adopted the reasonable interparticle distances of 2, 3, 5, and 7 nm, as shown in Figure 3f. More simulation details are provided in Note S3 (Supporting Information). The oxygen transport resistance is decreased by over 50% with increasing the Pt‐Pt interparticle distance from 2 to 5 nm, while changes a little with that from 5 to 7 nm. Therefore, >5 nm of Pt‐Pt interparticle distance can effectively migrate the co‐attraction effect of Pt particles, thereby avoiding the high oxygen transport resistance. The oxygen transport resistances to the 3 nm Pt particles with different interparticle distances are shown in Figure S10 (Supporting Information). The changing trend of oxygen transport resistance at different interparticle distances is the same, and >5 nm of Pt‐Pt interparticle distance is still essential to avoid high oxygen transport resistance. Moreover, the experimental results by Inaba et al.^[^ ^39^ ^]^ demonstrated that the ECSA of catalysts rapidly increased with the Pt‐Pt interparticle distance of <5 nm and kept constant with the one of >5 nm for the Pt particles of 1.8 nm. The ECSA represents the capability of catalysts in contact with oxygen molecules for reaction. A low ECSA results in a high oxygen transport resistance especially at high current densities.^[^ ^17^ ^]^ Therefore, Inaba's results effectively support our MD simulation results.

**Figure 4:**
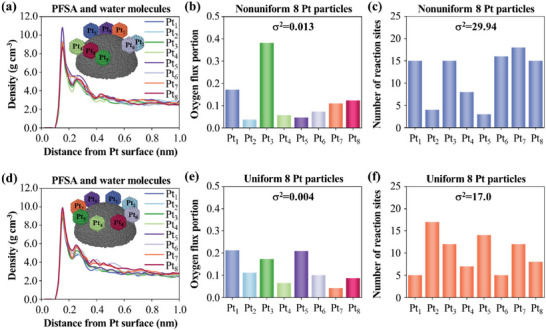
a,d) 1D density distributions of PFSA and water molecules, b,e) oxygen transport flux portions to each Pt particle, and d,f) number of reaction sites on each Pt particle that oxygen molecules can reach for nonuniformly a–c) and uniformly d–f) arranged Pt particles.

Furthermore, the oxygen transport near nonuniformly and uniformly arranged Pt particles is further analyzed. First, the total densities of PFSA and water molecules at different distances from each Pt particle surface for nonuniformly and uniformly arranged Pt particles are computed and plotted in **Figure** **4**a,d, respectively. For nonuniformly arranged Pt particles, the total density peaks near different Pt particles supported by an identical carbon particle show a pronounced difference as shown in Figure 4a, while for uniformly arranged Pt particles, those show nearly the same as shown in Figure 4d. Moreover, the oxygen transport fluxes to each Pt particle are obtained and plotted in Figure 4b,e, respectively. For nonuniform Pt particles, the Pt particles of Pt_1_ and Pt_3_ contribute 55.4% of total oxygen transport flux, while the other six Pt particles only contribute 44.6% as shown in Figure 4b. By contrast, for uniformly arranged Pt particles, the oxygen transport fluxes to each Pt particle show a smaller difference with less than one‐third of variance (*σ^2^*) in comparison with nonuniformly arranged Pt particles as shown in Figure 4e. Therefore, the uniform ionomer density on uniformly arranged Pt particles will improve the homogeneity of oxygen transport flux to each Pt particle. Moreover, the reaction sites on Pt particles that oxygen molecules can reach are counted for each Pt particle and shown in Figure 4c,f. The *σ^2^* of the reaction sites of each Pt particle is 29.94 for nonuniformly arranged Pt particles, which is nearly twice as high as that for uniformly Pt particles. Therefore, the uniform Pt particles can make the reaction site numbers on each Pt particle more consistent. The homogeneous oxygen transport fluxes and reaction sites may prevent Pt catalysts from degradation due to the severe reaction at a small number of reaction sites.

### 2.3 Arranging Pt Particles for Low Oxygen Resistance

**Figure 5:**
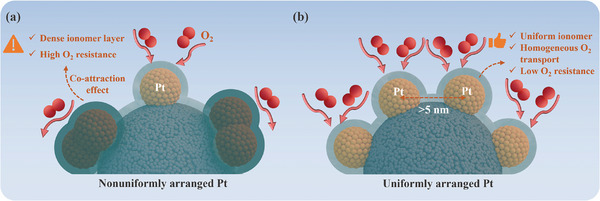
Schematics of ionomer film structures and oxygen permeation mechanisms for a) nonuniformly and b) uniformly arranged Pt particles on the amorphous carbon.

Local oxygen transport resistance is highly dependent on the arrangement of Pt particles on the carbon support, including the Pt particle dispersity and uniformity. Specifically, the small Pt‐Pt interparticle distance under a lower dispersity causes a high‐density ionomer layer due to the co‐attraction of Pt particles, thereby leading to a high local oxygen transport resistance as shown in **Figure** **5**a. After evaluation, >5 nm of Pt‐Pt interparticle distance is preferred to mitigate the co‐attraction effect and significantly decrease the local oxygen transport resistance as shown in Figure 5b. The non‐uniform arrangement of Pt particles on the carbon support inevitably leads to the small interparticle distance between some Pt particles, thereby resulting in the denser ionomer layer and higher local oxygen transport resistance near them as shown in Figure 5a. In contrast, the uniform arrangement of Pt particles with similar interparticle distance makes the ionomer layer density on them more homogeneous, and as a result, the local oxygen transport resistance through ionomer films on each Pt particle as well as the reaction site number of each Pt particle is more consistent as shown in Figure 5b. Therefore, to uniformly arranged Pt particles with an identical interparticle distance of >5 nm is preferred for reducing local oxygen transport resistance and improving the homogeneity of oxygen transport as shown in Figure 5b. Modifying the carbon support through optimizing surface defects and doping oxygen or nitrogen functional groups is an effective strategy for synthesizing an ideal arrangement of Pt particles on the carbon support. For example, Islam et al.^[^ ^10^ ^]^ introduced a modified carbon support with intrinsic nitrogen‐doping, which achieved the uniformly arranged and spacing‐controlled Pt particles on the carbon support.

## 3 Conclusion

Local oxygen transport resistance causes the severe voltage loss of low Pt PEMFCs, highly limiting the large‐scale commercialization of PEMFCs. A primary approach for reducing local oxygen transport resistance is to rationally arrange Pt particles on carbon support. This paper develops a well‐designed MD simulation procedure adopting the real amorphous carbon particle obtained by the HRMC method to investigate the critical role of Pt catalyst particle arrangement in local oxygen transport resistance. Experiments for obtaining local oxygen transport resistances are performed to verify the MD simulation. MD simulation results demonstrate that densely arranged Pt particles with a small interparticle distance strongly attract more PFSA chains to form a denser ionomer layer on them due to the co‐attraction effect. Under the attraction of Pt particles, the densities of PFSA and water on the carbon support decrease and increase, respectively, enhancing the hydrophilicity of the carbon surface when the Pt particle dispersity decreases. Both the simulated and experimental results show that the oxygen transport resistance increases with the decrease of Pt particle dispersity (i.e., decrease of Pt‐Pt interparticle distance), especially at a low dispersity. High oxygen transport resistance near Pt particles with a small interparticle distance is ascribed to the dense ionomer layer induced by the co‐attraction effect of Pt particles. We further evaluate the relationship between the oxygen transport resistance and Pt‐Pt interparticle distance. The results show that the oxygen transport resistance is reduced by over 50% when the Pt‐Pt interparticle distance is increased from 2 nm to 5 nm. Therefore, >5 nm of Pt‐Pt interparticle distance can effectively mitigate the co‐attraction effect, thereby preventing the high oxygen transport resistance. Moreover, the nonuniform arrangement of Pt particles inevitably causes some Pt particles with a small interparticle distance, and as a result, the ionomer layer on them is denser and the oxygen transport resistance becomes higher. In this case, the ionomer densities, oxygen transport resistances, and reaction site numbers near the Pt particles on carbon are heterogeneous. Therefore, to uniformly arranged Pt particles with a large interparticle distance of >5 nm is preferred for reducing local oxygen transport resistance and improving the homogeneity of oxygen transport, which is promising for the next‐generation low‐Pt PEMFCs.

## 4 Experimental Section

### Reconstruction of Amorphous Carbon

Hybrid Reverse Monte Carlo (HRMC) method^[^ ^40^, ^41^ ^]^ was used to fit the experimental diffraction structure and minimize the system energy to generate the amorphous carbon as the catalyst support. The potential energy between carbon atoms was represented by Environment‐Dependent Interatomic potential.^[^ ^42^ ^]^ During the reconstruction based on HRMC, a random configuration was first generated and then the carbon atoms following the specific constraint were continually moved to a new position until the error function keeps nearly constant. Specifically, the error function, *χ*, is defined as:
(1)

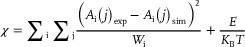

where *A*_exp_ and *A*_sim_ are the experimental and simulated cost function, respectively; *i* is defined as 1 or 2; *j* represents the number of discrete points in the experimental data; *W* is the weight factor of cost function; *E* is the total system energy; *K*_B_ is the Boltzmann constant; and *T* is the simulation temperature. The cost function *A* represents the structure factor function of *S*(*q*) when *i* = 1, while the radial distribution function of *g*(*r*) when *i* = 2. The *S*(*q*) represents the scattering ability of materials, which is affected by the atomic number, scattering direction, and atomic position. The structure factor of materials can be obtained via neutron diffraction, small‐angle X‐ray scattering, etc. The *g*(*r*) is the probability of finding the particle B at radius *r* from the particle A, which can be expressed as:

(2) $$g_{A-B}=\left(\frac{n_{B}}{4\pi r^{2}dr}\right)/\left(\frac{N_{B}}{V}\right)$$

where *n*_B_ is the number of B particles within the spherical shell of thickness *dr* at a radius *r* from the A particles, 4*πr* ^2^ *dr* represents the volume of the shell, *N*_B_ is the total number of B particles in the simulation system, and *V* is the total volume of the simulation system. In this study, the experimental *S*(*q*) is obtained from Walters's work,^[^ ^35^ ^]^ while the experimental *g*(*r*) is derived via the fast Fourier transform of *S*(*q*).^[^ ^40^ ^]^ The *χ* of a carbon atom was computed before and after its movement, represented by *χ*_old_ and *χ*_new_, respectively. If *χ*_new_ < *χ*_old_, the movement of this carbon atom was accepted. Otherwise, the probability of allowing moving this carbon atom is set to *P*, which is defined as follows.

(3) $$P=e^{\chi_{\mathrm{old}}-\chi_{\mathrm{new}}}$$

The HRMC simulation starts at a high temperature of 6000 K, and the temperature linearly decreases to 300 K in 40 million steps to obtain a stable amorphous carbon structure. Meanwhile, carbon atoms were continuously moved during the temperature decrease until the error function *χ* reached constant. More details of HRMC simulation are provided in Note S1 (Supporting Information). Finally, the comparison between the simulated and experimental *S*(*q*) in Figure 1a shows that the reconstructed amorphous carbon (Figure 1b) agrees with the real one. Moreover, the radial distribution function *g*(*r*), system temperature, and single atom energy are plotted in Figure S11 (Supporting Information).

### Molecular Models and Simulation Procedures

The schematics of amorphous carbon, Pt particle, water molecule, hydronium ion, and repeating monomer of PFSA are shown in Figure 1c. A hemispherical amorphous carbon with a radius of 4 nm is adopted considering its symmetrical characteristic, which contains 19460 carbon atoms. Previous studies have proved that the optimized Pt particle size is 2–4 nm for improving catalytic activity.^[^ ^39^, ^43^ ^]^ Therefore, the diameters of Pt particles are defined as 2 and 3 nm in this study. The PFSA chain contains 10 repeating monomers with 16 CF_2_ groups and a side chain.

The sizes of the simulation box are set as 9.2, 9.2, and 50 nm in the X, Y, and Z directions, respectively. The carbon support was placed in the center of the bottom surface of the simulation box. The number of Pt particles on the carbon support is set as 1, 2, 4, and 8, to represent different Pt particle dispersity of 0.83, 0.71, 0.55, and 0.38, respectively. The Pt particle dispersity *D* is calculated by:

(4) $$D=\frac{m_{c}}{m_{c}+m_{\mathrm{Pt}}}$$

where *m*_c_ and *m*_pt_ are the total weights of carbon and Pt particles, respectively.^[^ ^44^ ^]^ Moreover, multiple Pt particles are nonuniformly and uniformly placed on the carbon support, respectively, to represent different Pt particle uniformity as shown in Figure 1c. The center coordinates of the carbon support and each Pt particle are shown in Table S2 (Supporting Information). 30 PFSA chains, 300 hydronium ions, and 3000 water molecules were randomly inserted on carbon‐supported Pt particles to establish the original configuration as shown in Figure 1d. The water content, that is the number ratio of water molecules and hydronium ions to sulfonic acid groups in PFSA chains, is 11 in this study, which is the common one in the actual CL.^[^ ^45^, ^46^ ^]^ Afterward, a series of processes, including compressing, relaxing, annealing, and equilibrating processes, were executed to get an equilibrium ionomer film configuration covering carbon‐supported Pt particles. The thickness of the equilibrium ionomer film is ≈4 nm, which follows the actual ionomer thickness in the CL.^[^ ^47^ ^]^ More simulation details are provided in Note S2 (Supporting Information). Then, 1800 oxygen molecules were inserted into the region above the ionomer film, as shown in Figure 1e. As shown in Equation (6), Oxygen molecules reaching the Pt surface were removed, and new ones were added to the simulation box to simulate oxygen transport through ionomer films and consumption on catalysts.

The potential energy function between atoms is expressed as

(5) $$\begin{array}{ccc} E & = & \sum K_{b}{\left(b-b_{0}\right)}^{2}+\sum K_{\theta }{\left(\theta -\theta_{0}\right)}^{2}+\sum K_{d}\left[1+d\cos\left(n\phi \right)\right]\\ & & +\;\sum 4\varepsilon \left[{\left(\frac{\sigma }{r}\right)}^{12}-{\left(\frac{\sigma }{r}\right)}^{6}\right]+\sum C\frac{qq^{\prime }}{r} \end{array}$$

where the five terms on the right were the bond stretching potential, bond angle bending potential, torsion angles potential, van der Waals potential, and electrostatic interaction potential energy in turn. The corresponding parameters have been verified in the previous works.^[^ ^30^, ^48^ ^]^ All MD simulations were performed using the large‐scale atomic/molecular massively parallel simulator package.^[^ ^49^ ^]^

The oxygen transport flux through the ionomer film to Pt particles ($J_{O_{2}-\mathrm{Pt}}$, mol s^−1^ m^−2^) is defined as:

(6) $$J_{O_{2}-\mathrm{Pt}}=\frac{N_{O_{2}-\mathrm{Pt}}}{N_{A}At}$$

where $N_{O_{2}-\mathrm{Pt}}$ is the number of oxygen molecules reaching Pt particles, which is recorded every 2 ps and averaged every 3 ns; *N*_A_ is the Avogadro constant; *A* is the total surface area of Pt particles; and *t* is the simulation time. Then the oxygen transport resistance *R*_Local_ (s m^−1^) is calculated by:

(7) $$R_{\mathrm{Local}}=\frac{\Delta C_{O_{2}}}{J_{O_{2}-\mathrm{Pt}}}$$

where $\Delta C_{O_{2}}$ (mol m^−3^) is the oxygen concentration difference between the gas region and the Pt surface. The oxygen concentration at the Pt surface is ≈0 due to the electrochemical reaction. Moreover, as shown in Equations (6) and (7), a high oxygen concentration difference means a high oxygen transport flux, which will not affect the computed oxygen transport resistance.

### Experimental Procedures

The cathode CLs with different Pt particle dispersity were fabricated by spraying catalyst inks on the NR‐211 membrane, as shown in Figure 1f. The ultrasonic spray coating system used in this study is shown in Figure S12 (Supporting Information), and the material parameters during the CCM preparation are listed in Table S1 (Supporting Information). The catalysts of 20% Pt/C, 60% Pt/C, and 40% Pt/C (mixing the 20% Pt/C and 60% Pt/C by 1:1) were used in the cathode, which has the Pt particle dispersity of 0.8, 0.4, and 0.6, respectively. And all the anode catalysts are 60% Pt/C. The catalysts were mixed with the isopropanol and 5wt% of Nafion solution, followed by a ball‐milling for 24 h and ultrasonic dispersion for 1 h to obtain catalyst inks. The ball mill used in this work is shown in Figure S13 (Supporting Information). The mass ratio of ionomer and carbon is controlled at 0.8. The Pt loadings at the cathode and anode are both 0.1 mg cm^−2^. The microstructure of the cathode CL with a Pt dispersity of 0.4 via scanning electron microscope is shown in Figure S14 (Supporting Information).

Oxygen transport resistance is measured by the limiting current method^[^ ^27^, ^50^, ^51^, ^52^, ^53^ ^]^ The limiting current density (*i*_lim_) is the maximum current density when the oxygen concentration at the Pt surface approaches 0. Total oxygen transport resistance (*R*_Total_) at the cathode can be computed based on the *i*_lim_.

(8) $$R_{\mathrm{Total}}=\frac{4Fc_{O_{2}}}{i_{\lim}}=\frac{4F}{i_{\lim}}\frac{P_{O_{2}}}{RT}$$

where *F* is the Faraday constant; $c_{O_{2}}$ is the oxygen molar concentration in the flow field; $P_{O_{2}}$ is the oxygen partial pressure in the flow field; *R* is the gas constant, and *T* is the gas temperature. Physically, *R*_Total_ is divided into pressure‐dependent resistance (*R*_P_) and pressure‐independent resistance (*R*_NP_), which can be separated by measuring the *R*_Total_ under different operating pressures. The *R*_P_ is ascribed to Fick's diffusion of oxygen molecules in the flow field and large pores of porous electrodes, while *R*_NP_ is the Knudsen diffusion of oxygen molecules mainly in the small pores of CL and the oxygen permeation through ionomer films. In this study, only the Pt dispersity is adjusted in different CLs, which mainly affects the ionomer film structure covering carbon‐supported Pt particles. Therefore, the *R*_NP_ is obtained by calculating the intercept of *R*_Total_ to pressure, as shown in Figure S7 (Supporting Information), to evaluate the effect of Pt dispersity on the local oxygen transport resistance through ionomer films.

The area of the flow field plate in PEMFC is 1 cm^2^ as shown in Figure S15 (Supporting Information). The pure hydrogen and 2% oxygen diluted by nitrogen were adopted as the reactant at the anode and cathode, which are fed by the flow rates of 0.43 L min^−1^ and 1.05 L min^−1^, respectively. Moreover, the relative humidity and temperature are controlled at 100% and 70 °C, respectively. The inlet pressure is controlled as 160, 210, 260, and 306 kPa (absolute pressure) to obtain the curve between *R*_Total_ and *P*. The current density increases from 0.4 A cm^−2^ with a step of 0.016 A cm^−2^ by decreasing the voltage gradually to get the *i*_lim_.

## Conflict of Interest

The authors declare no conflict of interest.

## Supporting information



Supporting Information

## Data Availability

The data that support the findings of this study are available from the corresponding author upon reasonable request.
