# Supplementary material for: Molecular Understanding of the Role of Catalyst Particle Arrangement in Local Mass Transport Resistance for Fuel Cells
Source: Adv Sci (Weinh). 2024 Dec 15;12(5):2409755. doi: 10.1002/advs.202409755 (PMC11792038; doi:10.1002/advs.202409755)
Supplement: Supplementary file 1 — Supporting Information [file ADVS-12-2409755-s001.docx]

**Supporting Information**

**Molecular Understanding of the Role of Catalyst Particle Arrangement in Local Mass Transport Resistance for Fuel Cells**

Aoxin Ran^a,#^, Linhao Fan^a,b,^^c,#,*^, Chasen Tongsh^a,c^, Jiaqi Wang^a^, Zhengguo Qin^a^, Qing Du^a,*^, Meng Ni^b,*^, Kui Jiao^a,c,*^

a State Key Laboratory of Engines, Tianjin University, Tianjin, China

b Department of Building and Real Estate, Research Institute for Sustainable Urban Development (RISUD) & Research Institute for Smart Energy (RISE), Hong Kong Polytechnic University, Hong Kong, China

c National Industry-Education Platform for Energy Storage, Tianjin University, Tianjin, China

# Equal contribution

*Corresponding author: lhfan.tju.edu.cn (LF); meng.ni@polyu.edu.hk (MN); duqing@tju.edu.cn (QD); kjiao@tju.edu.cn (KJ)

Note S1. More details of HRMC simulation procedures

During the HRMC simulation, the sizes of the simulation box are set as 8nm, 8 nm, and 4nm in the X, Y, and Z directions, respectively. The number of carbon atoms is set as 37248, to form a density is 2.899 g cm^-3^. The structure factor function *S*(*q*) is discretized into *j*=401 data points in the range of 0-20, while the radial distribution function *g*(*r*) is discretized into *j*=1403 data points in the range of 0-19.9878. The *g*(*r*) is obtained by the Fast Fourier Transform (FFT) converted from *S*(*q*). The whole HRMC simulation includes four stages with the temperature changing from 6000 K to 3000 K, from 3000 to 1500 K, from 1500 to 300 K, and constant 300 K, each of which runs 10 million steps, as shown in Figure. S1b. The weight factor *W*_1_ of *S*(*q*) is equal to *W*_2_ of *g*(*r*), which are 0.016, 0.008, 0.004, and 0.001 for the four stages, respectively. After that, a stable configuration of amorphous carbon is obtained. Finally, a hemispherical structure in the original amorphous carbon is selected in this study, while the other atoms are deleted to reduce the calculational cost.

Note S2. More details of MD simulation procedures

The energy minimization is first performed after obtaining the initial configuration. Then a dummy potential wall is placed at the top of the simulation box, which interacts with atoms following the L-J function.

$E_{wall}=\left\{\begin{matrix} 4\varepsilon_{wall}[(\frac{\sigma_{wall}}{r_{wall}})^{12}-(\frac{\sigma_{wall}}{r_{wall}})^{6}] r_{wall}<0.25 nm \\ 0 r_{wall} >0.25 nm \end{matrix}\right.$ (S1)

where *r*_wall_ is the distance between atoms and the potential wall, and *ε*_wall_ and *σ*_wall_ are set as 0.01 kcal mol^-1^ and 0.25 nm, respectively. During the compressing process, the potential wall is moved along the thickness direction of ionomer film until the density of ionomer film reaches the specific value, which is the density of bulk membrane [S1]. During the relaxing process, 1 ns NVT simulation is performed at 1000 K using the L-J potentials within PFSA molecules and between Pt/C atoms and other atoms reduced by 2 orders of magnitude. Then 1 ns NVT simulation is performed with returning the L-J potentials to the initial values and the temperature to 353 K to eliminate the initial bias. During the annealing process, the system temperature is changed between 1000 K and 353 K for 8 times at a 1 ns NVT simulation. Afterward, the potential wall is lifted to ∼13 nm from the bottom wall of the simulation box. During the equilibrating process, another 20 ns NVT simulation is performed to make the configuration reach equilibrium. Furthermore,1800 oxygen molecules are inserted into the gas region above the equilibrium ionomer film. A total of 25 ns NVT simulation is conducted to simulate the oxygen permeation process when the data of the last 15 ns simulation are collected for analysis.

Note S3. MD simulations for different distances between Pt particles

To investigate the effect of the distance between Pt particles on the oxygen transport resistance, four distances of 2, 3, 5, and 7 nm between Pt particles are adopted. In this simulation, the carbon support is considered as a multilayer graphene with a size of 15 nm×7.5 nm, which supports two Pt particles. 38 PFSA molecules, 380 hydrated hydrogen ions, and 3800 water molecules are inserted into the region on carbon-supported Pt particles to establish the original configuration. Then a series of processes, including compressing, relaxing, annealing, and equilibrating processes, are executed to get an equilibrium configuration, followed by the oxygen permeation process, which is the same as those provided in Note S2.

Table S1 Material parameters during the CCM preparation

| **Material** | **Parameter** | **Manufacturer** |
| --- | --- | --- |
| Pt/C | 60 wt%, TEC10E60TPM | TANAKA |
| Pt/C | 40 wt%, TEC10E40E | TANAKA |
| Pt/C | 20 wt%, TEC10E20E | TANAKA |
| isopropanol | Purity: 99.5% | Aladdin |
| Nafion solution | 5 wt% | Dupont |
| NR-211® membrane | 25.4μm, 0.1 S cm-1 | Dupont |

Table S2. Center coordinates of carbon support and each Pt particle

|  | **Nonuniform distribution** | **Uniform distribution** |
| --- | --- | --- |
| Carbon support | (0.00,0.00,0.00) | (0.00,0.00,0.00) |
| Pt_1_ | (-0.85,39.75,36.87) | (17.85,66.67,33.51) |
| Pt_2_ | (60.25,60.25,30.50) | (46.45,54.30,33.51) |
| Pt_3_ | (53.15,-1.35,30.67) | (17.85,-2.37,33.51) |
| Pt_4_ | (4.95,2.75,30.00) | (46.45,-2.37,33.51) |
| Pt_5_ | (8.15,16.00,32.00) | (-2.37,46.45,33.51) |
| Pt_6_ | (64.85,40.15,37.10) | (66.67,74.52,33.51) |
| Pt_7_ | (26.15,67.15,35.20) | (-2.37,17.85,33.51) |
| Pt_8_ | (33.15,-2.95,35.70) | (66.67,17.85,33.51) |


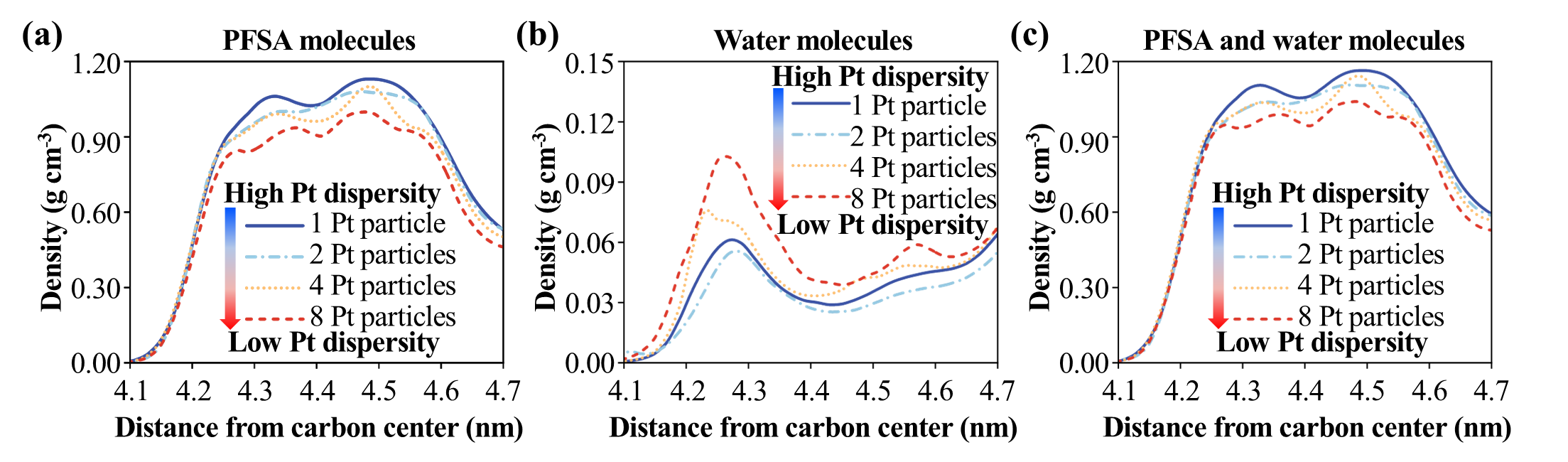


Figure. S1. One-dimensional density distributions of (a) PFSA molecules, (b) water molecules, (c) PFSA and water molecules at different distances from the carbon center for nonuniformly arranged Pt particles.


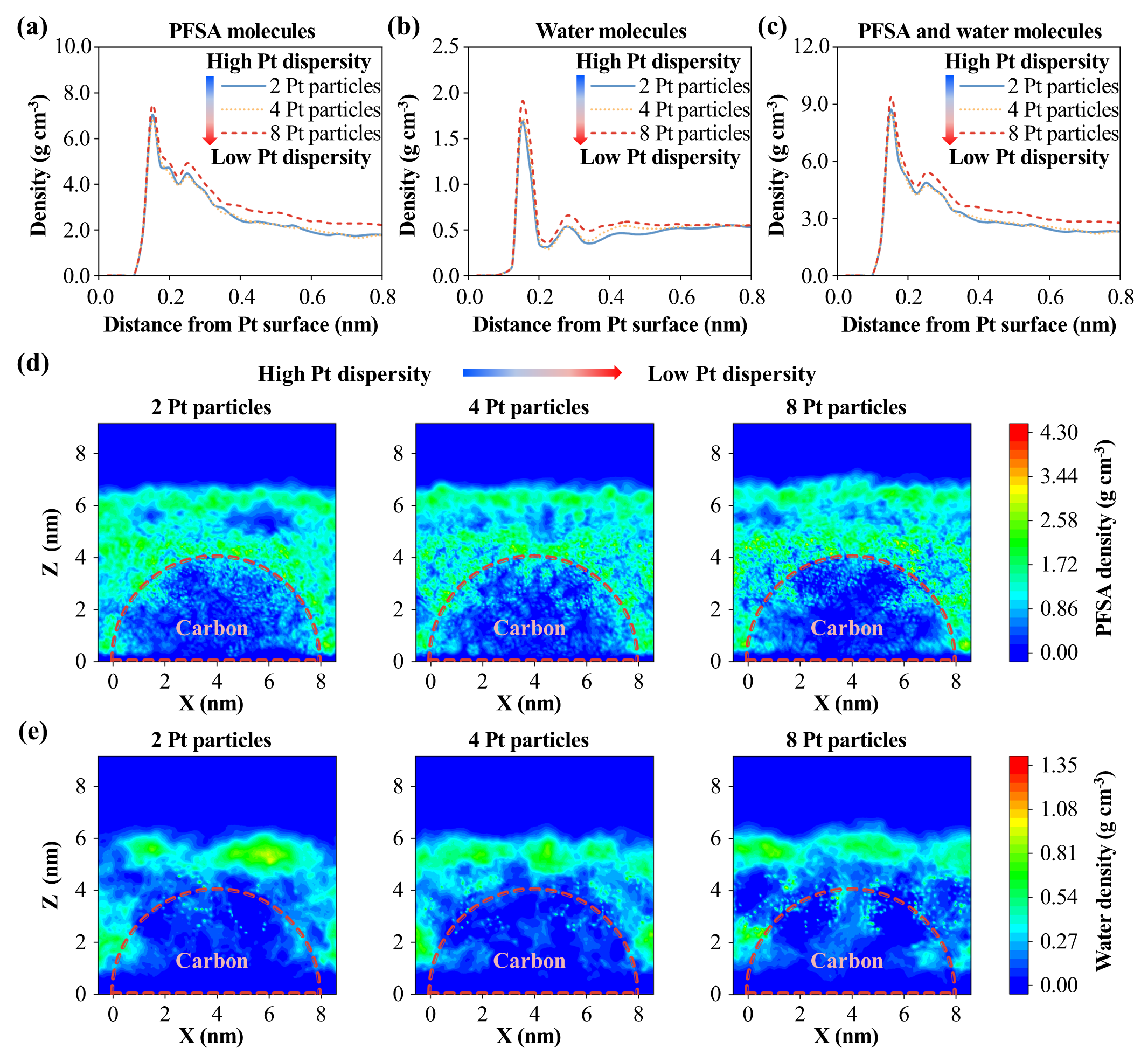


Figure. S2. One-dimensional density distributions of (a) PFSA molecules, (b) water molecules, and (c) all molecules at different distances from the Pt surface, and two-dimensional density distributions of (d) PFSA molecules and (e) water molecules on the X-Z plane for uniformly arranged Pt particles.


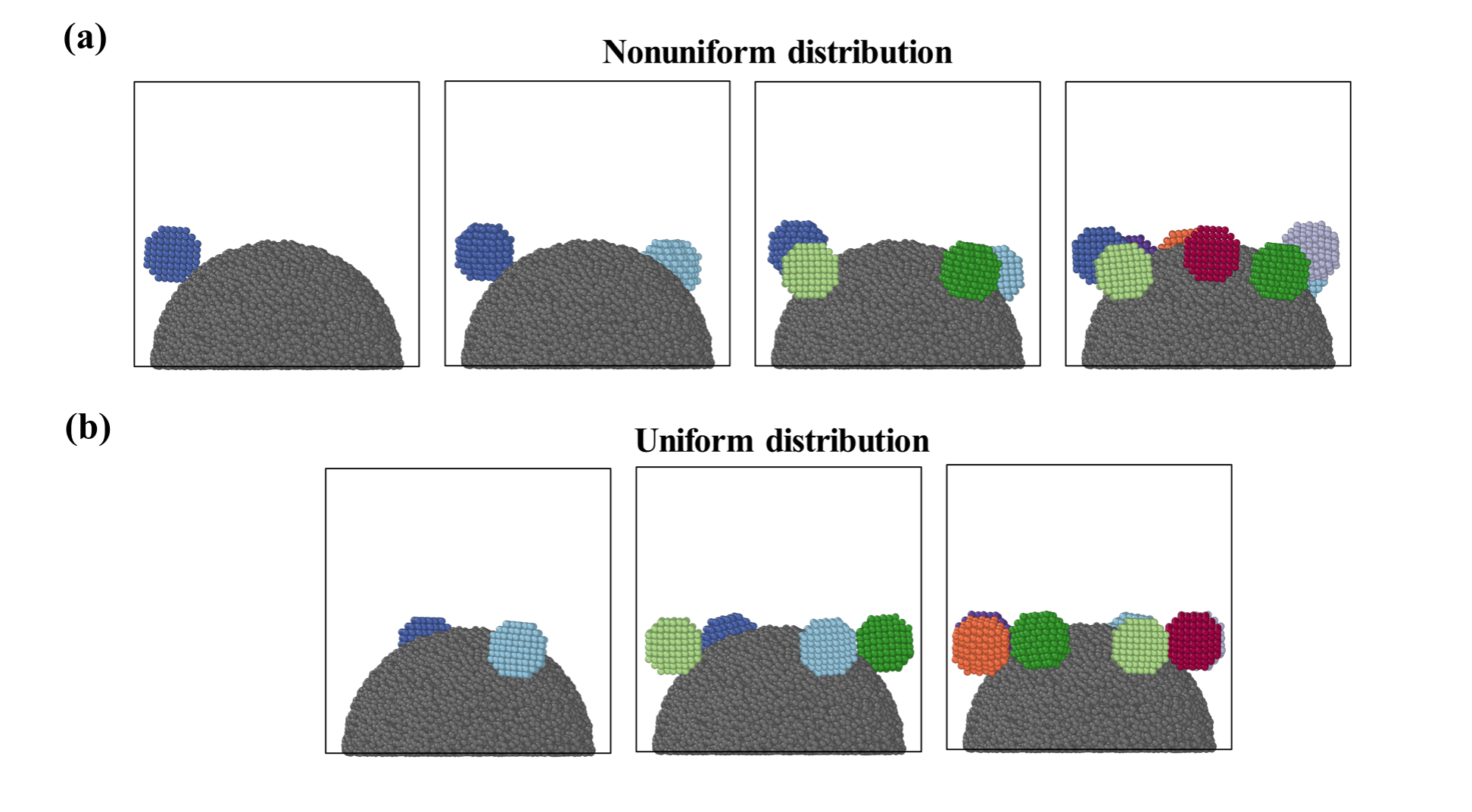


Figure. S3. Snapshots of Pt particles and carbon support for the (a) nonuniform Pt distribution and (b) uniform Pt distribution at different Pt dispersity.


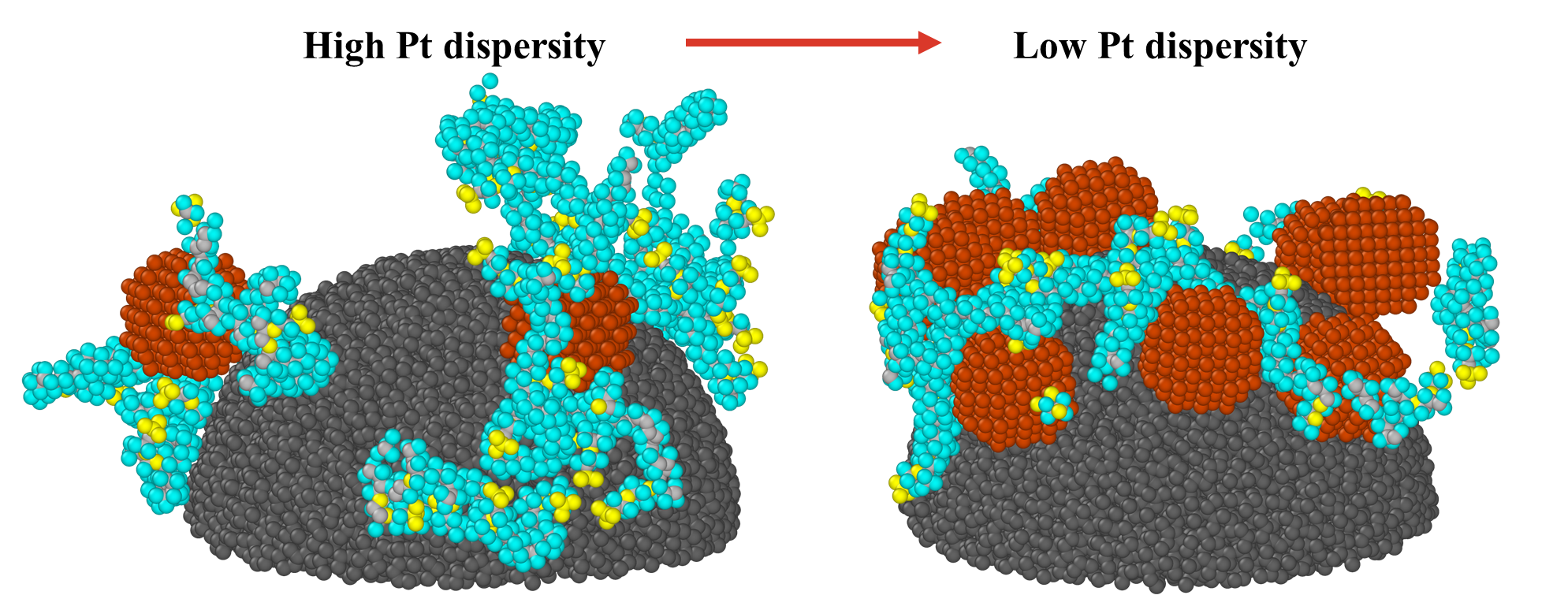


Figure. S4. Snapshots of PFSA molecules on carbon-supported Pt particles with a nonuniform distribution. The gray, cyan, yellow, blue, red, orange, and black beads represent the carbon atoms of PFSA, fluorine atoms of PFSA, sulfur/oxygen atoms of PFSA, water molecules/hydroniums, oxygen molecules, Pt atoms, and carbon atom of carbon support, respectively.


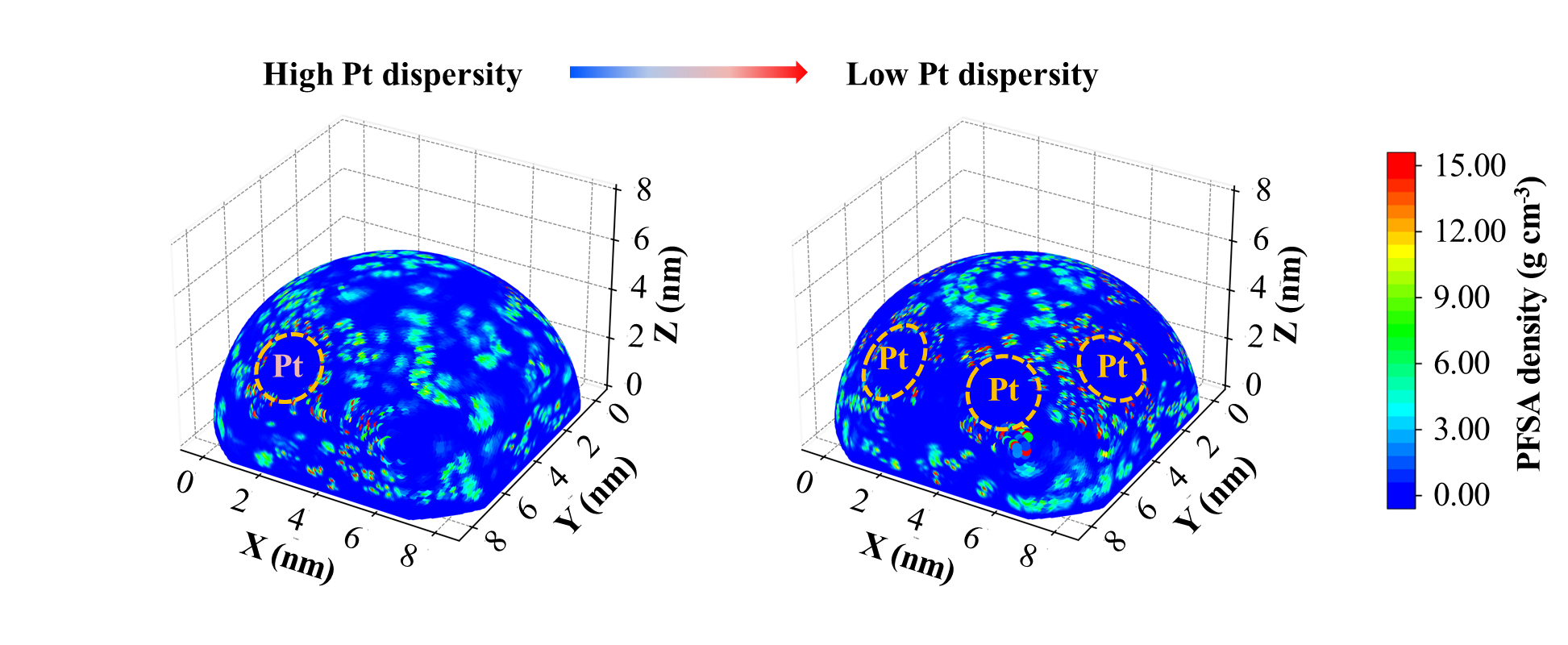


Figure. S5. Three-dimensional density of PFSA molecules near uniformly arranged Pt particles.


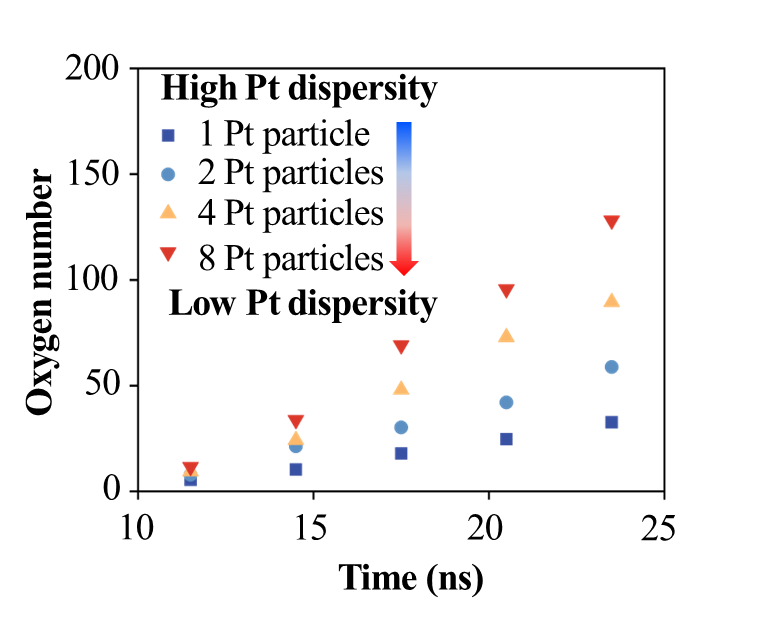


Figure. S6. Number of oxygen molecules that reach Pt particles at different simulation time for nonuniformly arranged Pt particles.


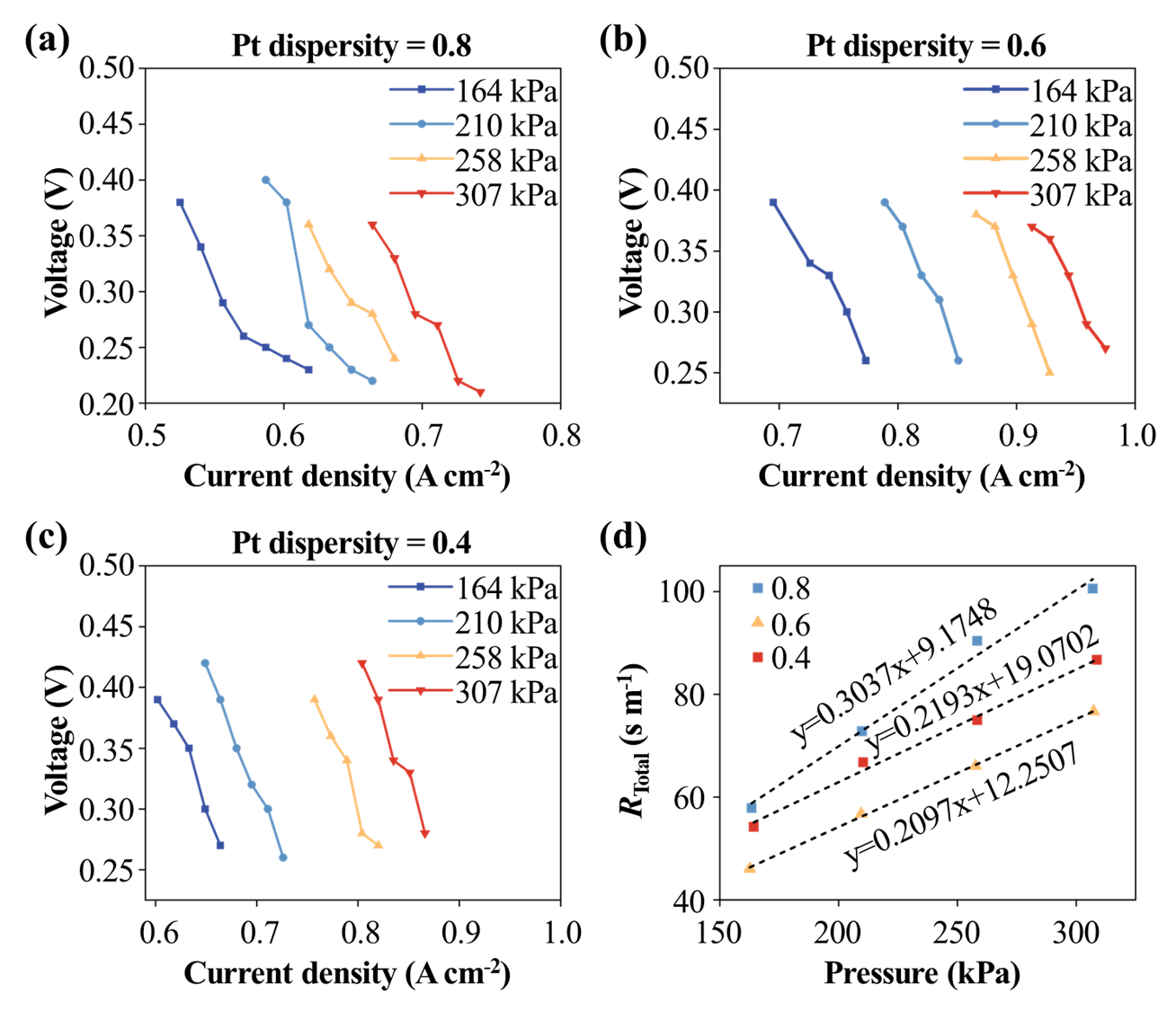


Figure. S7. Current-voltage curves in limiting current testing at the dispersity of (a) 0.8, (b) 0.6, and (c) 0.4, and (d) pressure-*R*_Total_ curve.


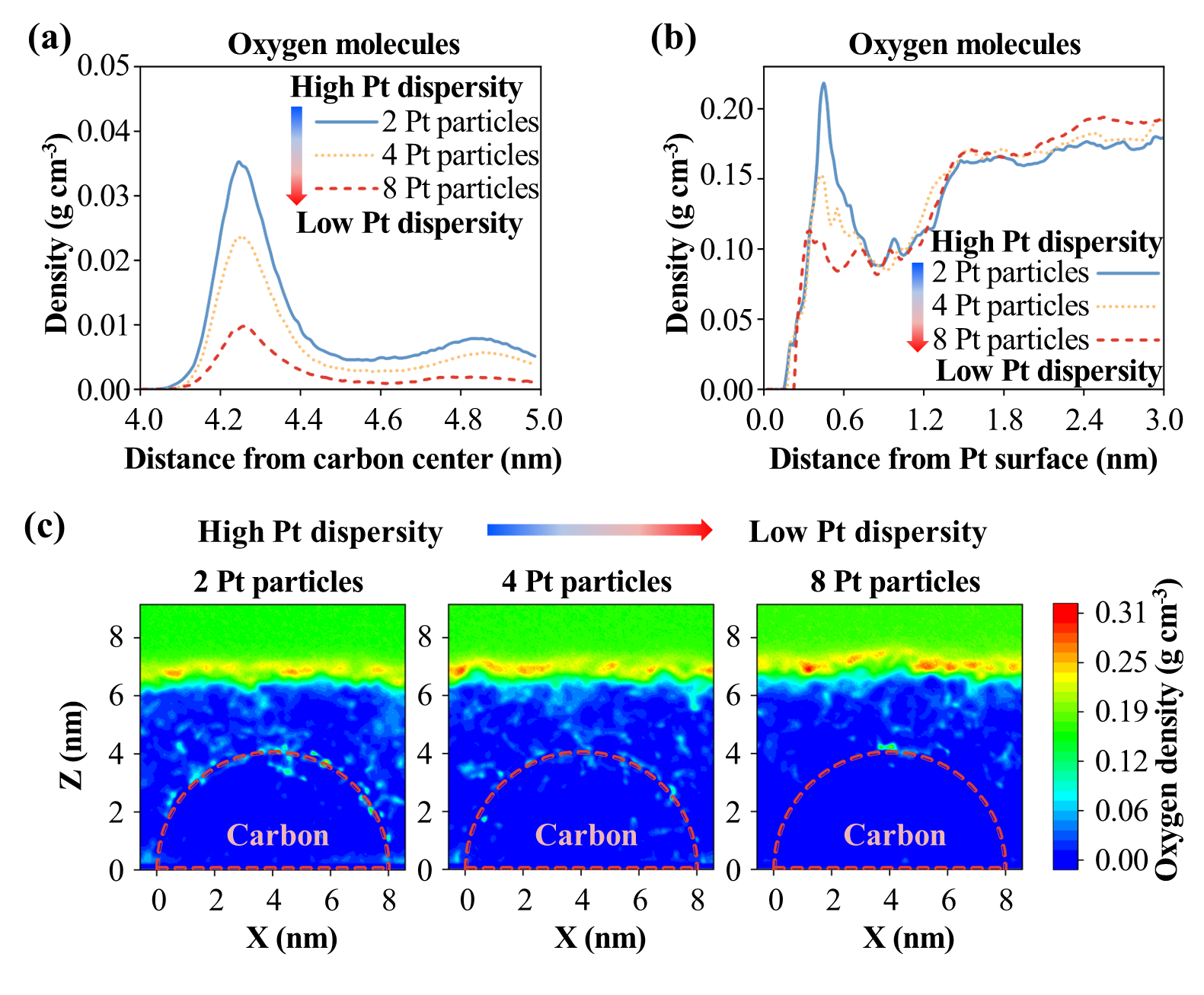


Figure. S8. One-dimensional density distributions of oxygen molecules at different distances from the (a) carbon center and (b) Pt surface, and (c) two-dimensional density distributions of oxygen molecules on the X-Z plane for uniformly arranged Pt particles.


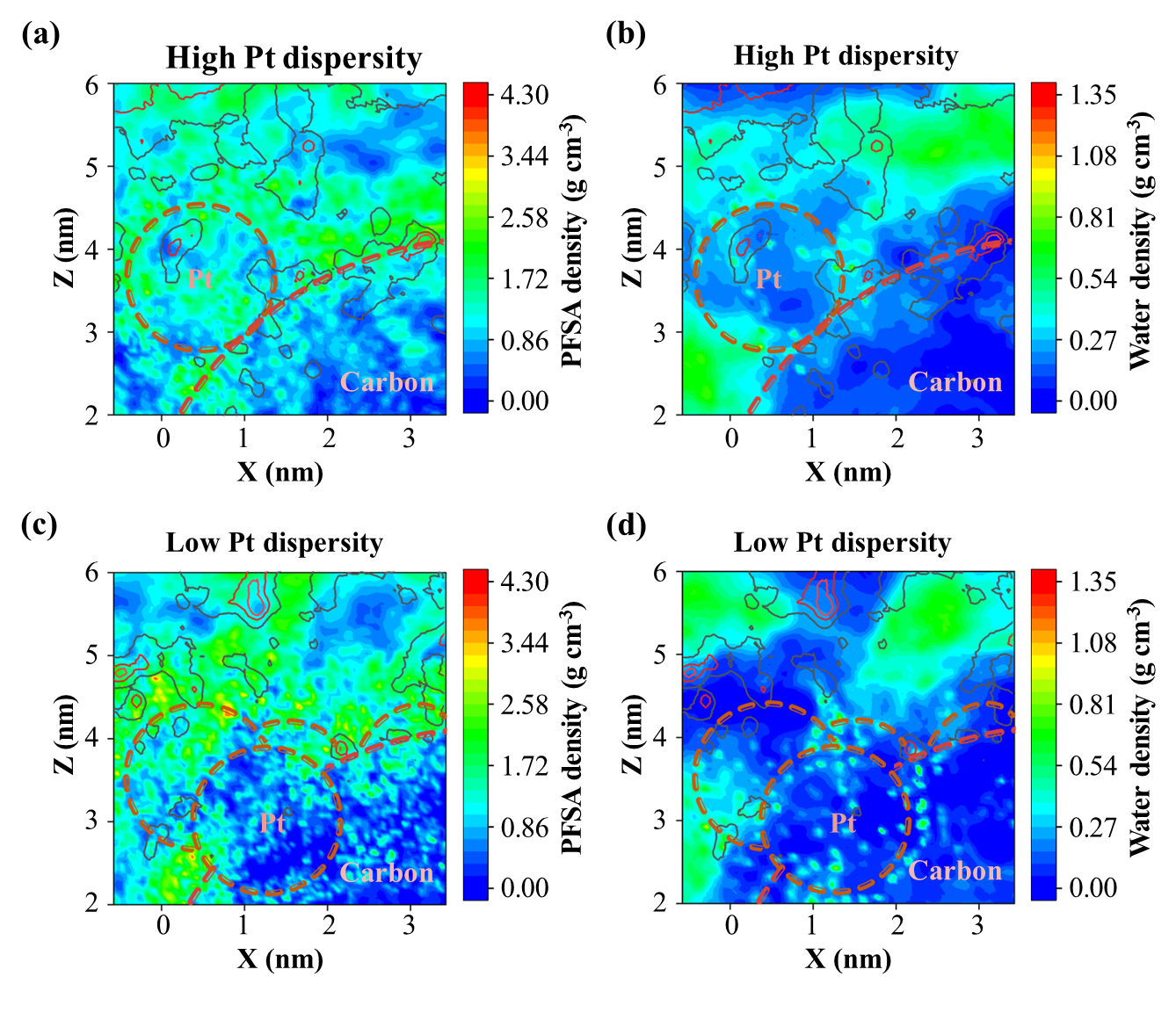


Figure. S9. Two-dimensional density distributions of (a and c) PFSA molecules and (b and d) water molecules. The black and red lines are the oxygen density contours at 0.04 and 0.1 g cm^-3^, respectively. The red dotted lines display the location of Pt particles.


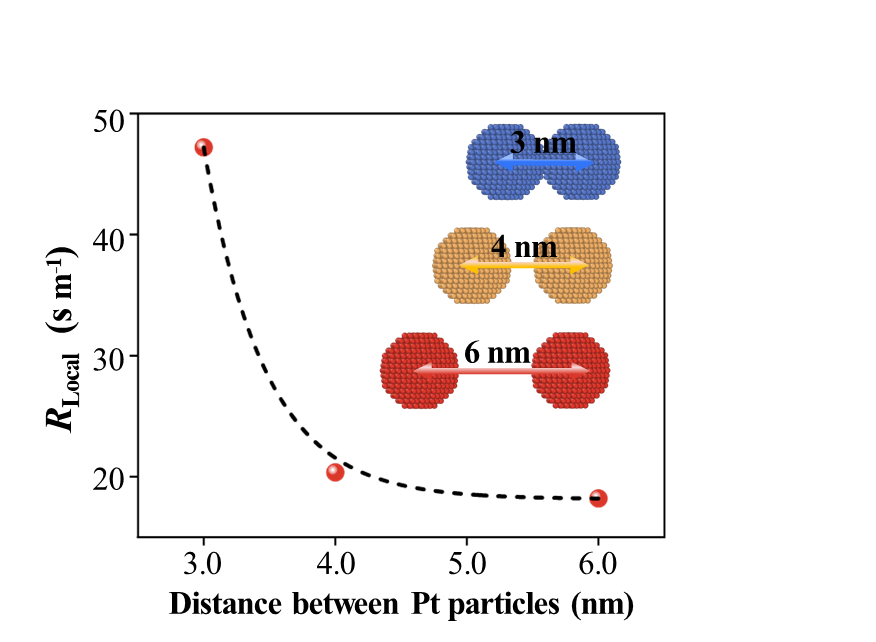


Figure. S10. Oxygen transport resistances (*R*_Local_) to Pt particles with different interparticle distances for the Pt particles of 3 nm.


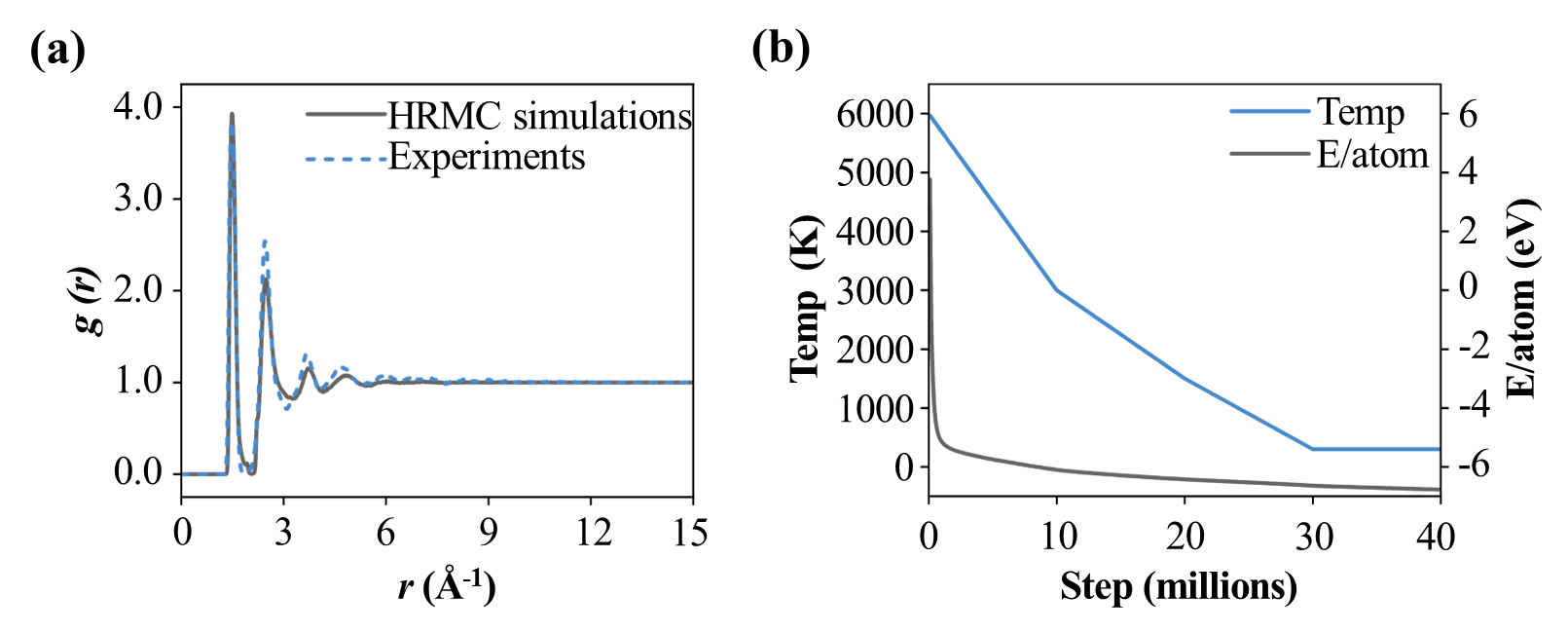


Figure. S11. (a) Radial distribution function *g*(*r*) of amorphous carbon obtained by HRMC simulations and experiments [S2], and system temperatures and atom energy during the quenching process.


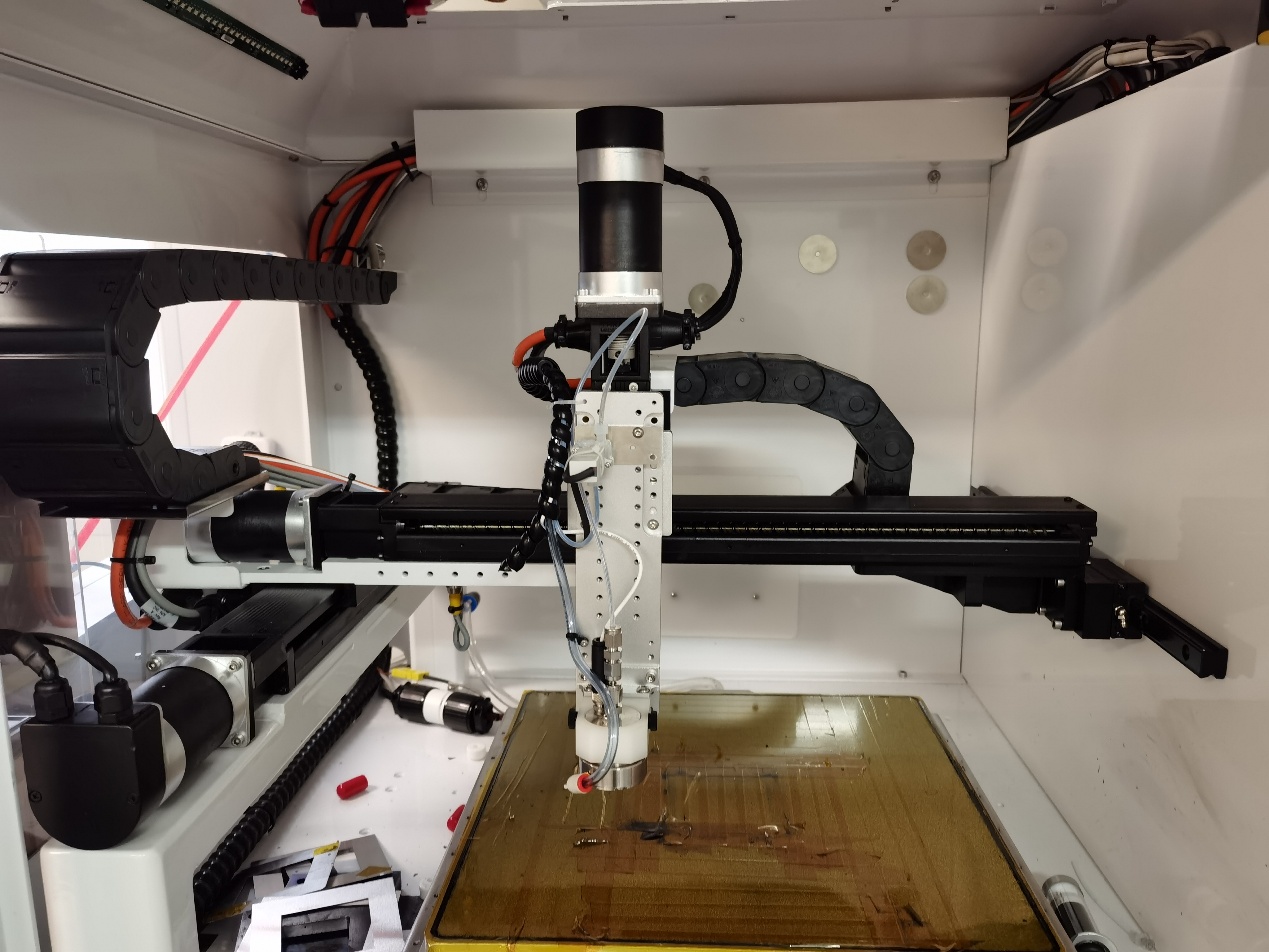


Figure. S12. Ultrasonic spraying instrument used to prepare the CL.


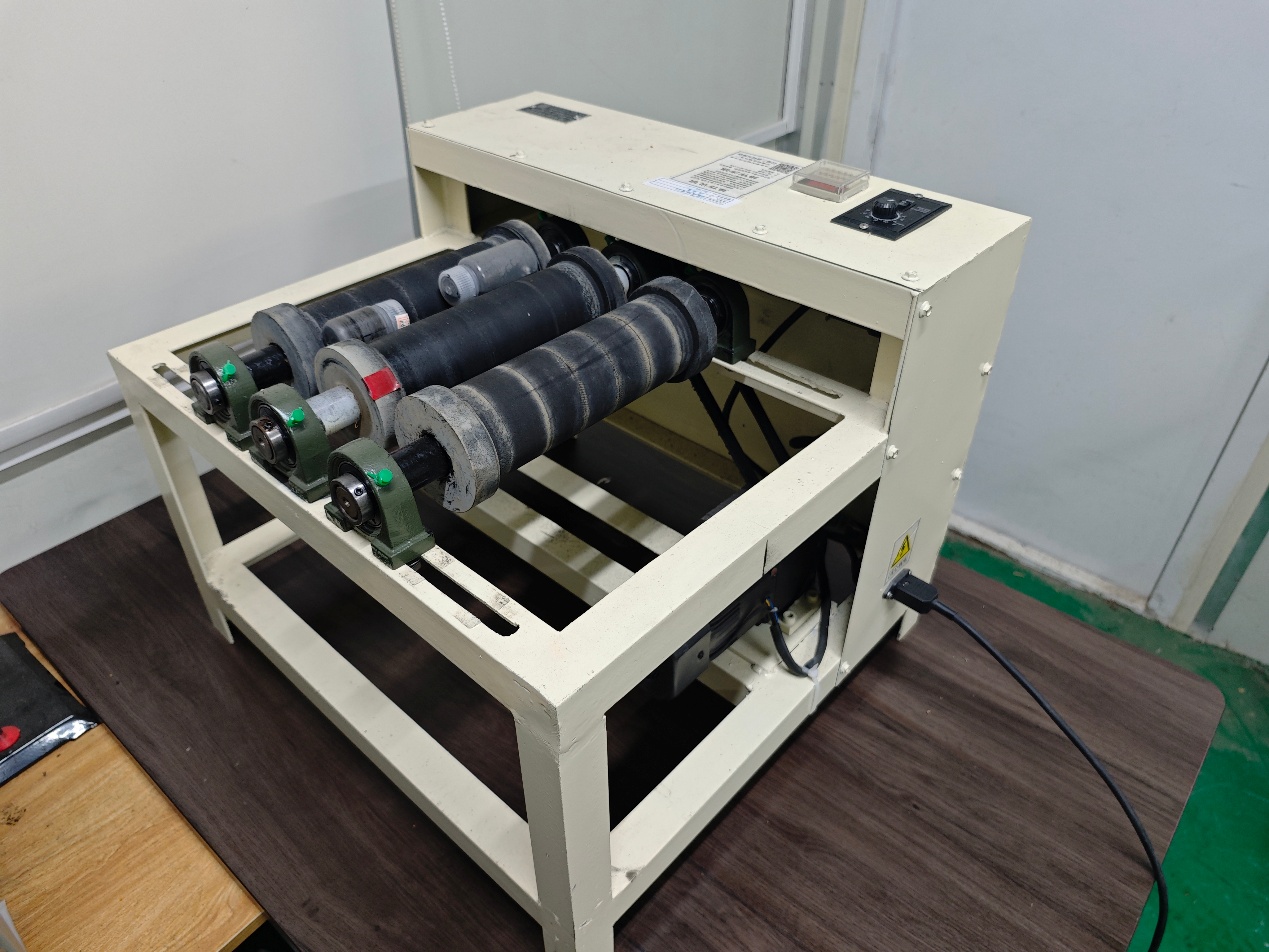


Figure. S13. The ball mill used to prepare the catalyst ink.


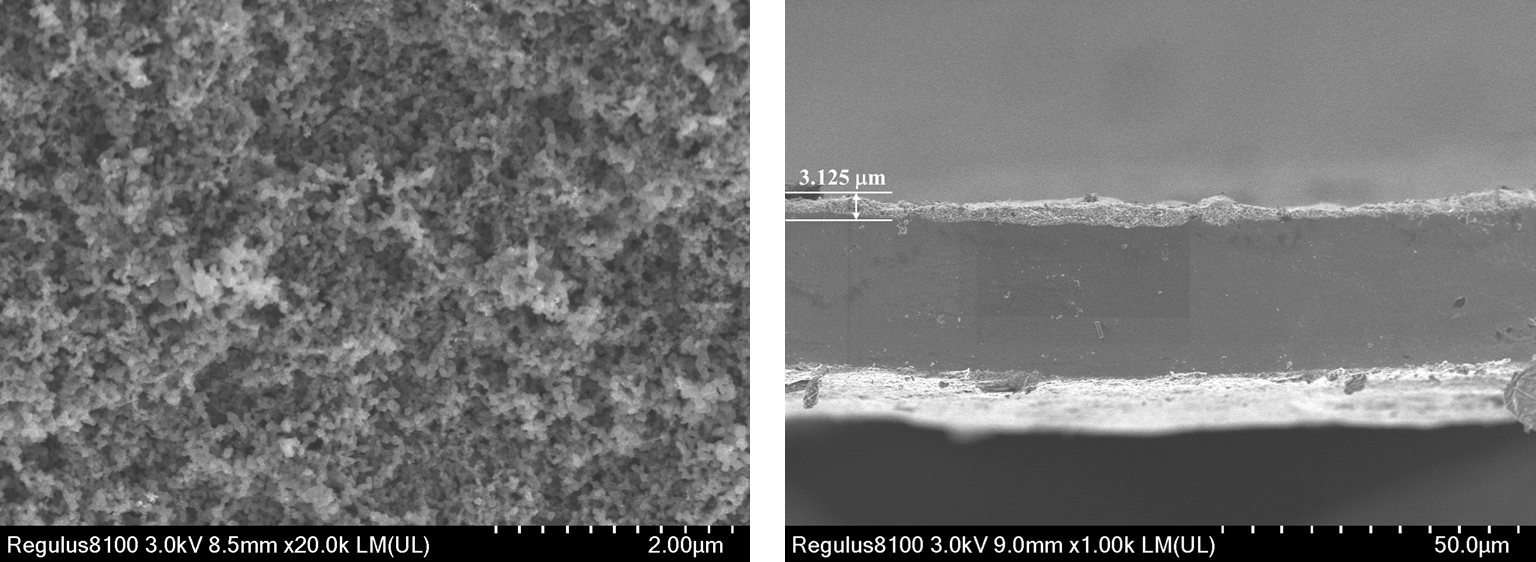


Figure. S14. SEM images of cathode CL with a dispersity of 0.6.


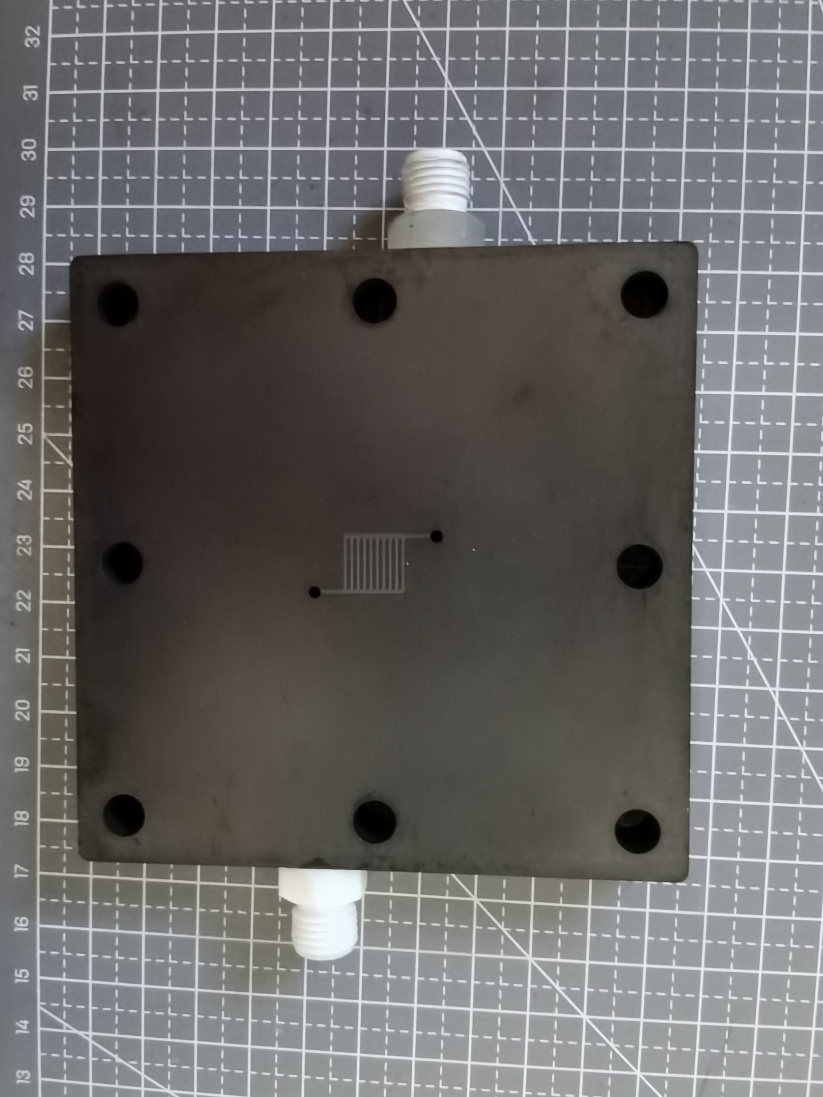


Figure. S15. Flow field plate with a surface area of 1.0 cm^2^ used for limiting current testing.

**References**

1. Fan L, Wang Y, Jiao K. Oxygen Permeation Resistances and Routes in Nanoscale Ionomer Thin Film on Platinum Surface. Journal of the Electrochemical Society 2021;168:014511.
2. Walters JK, Gilkes R, Wicks JD, Newport RJ. Progress in modeling the chemical bonding in tetrahedral amorphous carbon. Physical Review 1998;58:8267–76.
